# Correlation between job burnout, psychological status, and job satisfaction among anesthesiologists in the post-COVID-19 era: a cross-sectional study in China

**DOI:** 10.3389/fpubh.2025.1555141

**Published:** 2025-06-17

**Authors:** Xuemeng Chen, Rui Zhou, Xianjie Zhang, Jia Han, Feng Ju, Yukai Zhou, Leqiang Xia

**Affiliations:** ^1^Department of Anesthesiology, Deyang People’s Hospital, Deyang, Sichuan, China; ^2^Department of Anesthesiology and Perioperative Medicine, Shanghai Fourth People’s Hospital, School of Medicine, Tongji University, Shanghai, China

**Keywords:** job burnout, psychology, satisfaction, anesthesiologist, post-COVID-19 era

## Abstract

**Background:**

Burnout is a prevalent issue among healthcare professionals, particularly anesthesiologists, with significant repercussions for patient safety, personal well-being, and institutional efficiency. The post-COVID-19 era has introduced additional psychological and socioemotional stressors, which individuals perceive differently. This study aims to assess burnout levels among anesthesiologists in this era, explore its relationship with psychological status and job satisfaction, and propose potential intervention strategies.

**Methods:**

Four hundred electronic questionnaires were distributed to anesthesiologists licensed in 2024 with over 1 year of clinical experience. Three hundred twenty-six responses were collected, yielding an 81.5% response rate. The survey instruments included the Maslach Burnout Inventory-Human Services Survey (MBI-HSS), The Depression, Anxiety and Stress Scale – 21 Items (DASS-21), and Minnesota Satisfaction Questionnaire-Short Form (MSQ-SF). Descriptive statistics and adjusted linear regression were employed to analyze the data and examine the relationships between burnout, psychological status, and job satisfaction.

**Results:**

The study revealed a high prevalence of burnout, with 30.67% of participants at high risk and 24.85% exhibiting burnout syndrome, resulting in an overall burnout rate of 55.52%. Demographic factors were significantly associated with burnout (*p* < 0.05). Anesthesiologists aged 30–34 reported higher levels of anxiety and depression, while those aged over 35 showed lower levels of depersonalization and higher personal accomplishment (*p* < 0.001). Job satisfaction was inversely correlated with burnout (*p* < 0.001). Psychological status was positively correlated with burnout (p < 0.001). Multiple linear regression analysis explained 55.00% of the variance in burnout, with anxiety, stress, and intrinsic and extrinsic satisfaction as significant predictors (*p* < 0.05).

**Conclusion:**

Burnout is widespread among anesthesiologists in the post-COVID-19 era, with burnout levels strongly associated with mental health and job satisfaction. Increased negative emotions and reduced job satisfaction contribute to higher burnout. Addressing the well-being of anesthesiologists, fostering a supportive work environment, and improving compensation mechanisms could alleviate burnout and enhance the quality of medical practice.

## 1 Introduction

In China, there is a relative shortage of anesthesiologists, which is far lower than the number of anesthesiologists per 10,000 people in European and American developed countries (1). At the same time, the anesthesiologist is one of the most stressful positions in the medical industry. Anesthesiologists face unique work pressures, including long hours, high-risk decision-making, and high concentration during surgery, which make this special group bear severe psychological pressure and face all kinds of psychological problems. A survey showed that 15.3% of Chinese anesthesiologists showed high emotional exhaustion (2). Chinese anesthesiologists work extended hours per week and are at high risk of sudden death (2, 3). The post-COVID-19 era has reshaped socio-environmental dynamics, marked by increased employment pressures, fluctuating psychological anxiety levels, diverging employment confidence, heightened vulnerability to mental fragility, shifts in employment values (4–6). However, the specific mechanisms linking occupational burnout, mental health status, and job satisfaction among anesthesiologists in this context remain unclear. This study aims to investigate the correlations and their magnitudes between occupational burnout, psychological states, and job satisfaction among Chinese anesthesiologists in the post-COVID-19 era, providing novel theoretical support for alleviating occupational stress and enhancing workplace well-being among healthcare professionals.

## 2 Materials and methods

### 2.1 Design

This cross-sectional survey assessed the correlation of burnout, psychological status, and job satisfaction among anesthesiologists. This article adheres to the applicable Enhancing the QUAlity and Transparency of Health Research (EQUATOR) guidelines. Ethical approval for this study was obtained from the Ethics Committee of Deyang People’s Hospital (2024–04-012-K01). Clinical trial registration (ChiCTR2400082193) and written informed consent of the subjects were completed.

### 2.2 Population

Our target population was anesthesiologists who were already certified as practicing physicians and had been in clinical practice for more than 1 year. Sample size calculations based on anticipated response rates and confidence intervals. With an assumed prevalence (p) of 75% (7), a margin of error (*δ*) set at 0.05, and a Type I error rate (*α*) of 0.05, corresponding to a z-score of 1.96, the calculated sample size required was 289. Accounting for a 10% non-response rate, the necessary sample size was adjusted to 322. We further increased the sample size to accommodate potential refusals and invalid questionnaires. This study mainly distributed questionnaires via the WeChat Wenjuanxing network platform. Four hundred questionnaires were distributed, yielding 333 returns, of which 326 were valid, resulting in an effective response rate of 81.50%. The anesthesiologists in this questionnaire survey were mainly recruited from Sichuan province and a few from Shanghai, Guangdong, and Shandong. 79.1% (*n* = 258) were from tertiary hospitals, 17.2% (*n* = 56) were from tertiary hospitals, and 3.7% (*n* = 12) were from secondary hospitals, reflecting the hierarchical medical system in China. Moreover, compared with the national anesthesiologist workforce (8), the study cohort showed a higher proportion of postgraduate education and junior physicians.

### 2.3 Survey questions

#### 2.3.1 Basic characteristics of the population

The first part of the questionnaire contains 10 questions, which aims to understand the demographic, social, and work characteristics of anesthesiologists, such as gender, age, education, marital status, hospital level, professional qualifications, whether they hold administrative function, employment form, working hours per week, and whether they undertake teaching tasks.

#### 2.3.2 Maslach Burnout Inventory-Human Services Survey (MBI-HSS)

The Maslach Burnout Inventory-Human Services Survey (MBI-HSS) has become the gold standard for assessing burnout in health-related fields. This study adopted the Chinese version of the scale previously validated among Chinese physicians (3). The internal consistency reliability, measured by Cronbach’s *α*, was 0.929 in the current study, supporting its applicability to Chinese anesthesiologists. It assesses three dimensions of burnout -emotional exhaustion (EE), depersonalization (DP), and personal accomplishment (PA). EE measures feelings of emotional overactivity and exhaustion from work. DP refers to the provider adopting a cold and impersonal response to the patient. PA measures a person’s sense of competence and success at work. The MBIHSS consists of 22 questions, of which nine assess EE, five assess DP, and eight assess PA. Subjects provided answers on a 7-point Likert scale (using integer 0–6 codes) (9). In this study, individuals with EE (≥27) and/or DP (≥10) were classified as “High Risk for Burnout,” as these thresholds are established predictors of burnout development risk, even when personal achievement (PA) remains unaffected (7). Those meeting the “High Risk for Burnout” criteria and exhibiting PA scores ≤33 were further categorized as “Burnout Syndrome.” This classification aligns with prior burnout research and is consistent with definitions proposed by the World Health Organization (Geneva, Switzerland) and Maslach et al. (7, 9, 10). Differentiating these groups is critical for clarifying the progressive stages of burnout and providing evidence-based guidance for targeted policy interventions..

#### 2.3.3 Depression, Anxiety and Stress Scale – 21 Items (DASS-21)

The Depression, Anxiety and Stress Scale – 21 Items (DASS-21) is a self-report scale used to assess depression, anxiety, and stress levels in adults. This study utilized the validated Chinese version of the Depression, Anxiety, and Stress Scale-21 (DASS-21) (11). The internal consistency reliability, as measured by Cronbach’s *α*, was 0.921 in the current study, demonstrating good reliability and validity and confirming its applicability to the target population of Chinese anesthesiologists. It contains 21 items divided into three subscales of seven, each corresponding to depression, anxiety, and stress, with respondents rating each item on a scale of 0 to 3. The total score for each subscale ranged from 0 to 2 (12, 13).

#### 2.3.4 Minnesota Satisfaction Questionnaire-Short Form (MSQ-SF)

The Minnesota Satisfaction Questionnaire-Short Form (MSQ-SF) is a psychological tool that assesses employees’ satisfaction with various aspects of their jobs. It contains 20 items involving three dimensions: intrinsic job satisfaction (sense of achievement, recognition), extrinsic job satisfaction (such as pay and working conditions), and overall job satisfaction. This study employed the validated Chinese version of the Minnesota Satisfaction Questionnaire Short Form (MSQ-SF) (14). The internal consistency reliability, as measured by Cronbach’s *α*, was 0.916, demonstrating robust reliability and validity, confirming its suitability for the target population in this research. Respondents rated each item according to their level of agreement, usually on a 5-point Likert scale, with higher scores indicating greater satisfaction (15).

### 2.4 Procedures

The electronic version of the questionnaire for this study was produced and distributed through the Questionnaire Star platform^1^, utilizing WeChat, a widely used social platform in China, to ensure that the questionnaire could reach a wide range of respondents. The distribution cycle of the questionnaire was set as August 2024 for 1 month. During this period, we promoted through WeChat and other social media channels and set up regular reminders to increase the recovery rate.

Our recruitment procedures emphasized anonymity and voluntary participation to maintain data quality and mitigate selection bias. We applied two stringent criteria when screening the returned questionnaires. We applied two stringent criteria when screening the returned questionnaires. We proactively excluded any questionnaires completed in less than 90 s, suspecting the respondents’ lack of engagement or attention. Additionally, we discarded questionnaires where the same answer was selected for eight or more consecutive questions, indicating a mechanical or inattentive completion process.

### 2.5 Statistical analysis

Data analysis was performed using SPSS (version 29; IBM, Armonk, NY). Descriptive statistics were calculated as percentages for binary and categorical data, as means and standard deviations for normally distributed continuous variables, or as medians and interquartile ranges (IQR) for skewed continuous variables. Descriptive analysis used the number of cases and percentage to describe general data (such as gender, age, education, and professional qualification) and compared the scores of job burnout, psychological state, and job satisfaction under specific demographic characteristics. Independent sample *t*-test or one-way analysis of variance was performed. Pearson correlation analysis was used to investigate the correlation between job burnout, psychological status, and job satisfaction. A multiple linear regression model was constructed with occupational burnout as the dependent variable, including dimensions of the Depression, Anxiety, and Stress Scale-21 (DASS-21) and job satisfaction as independent variables while adjusting for covariates: age, education level, marital status, hospital tier, professional qualifications, administrative role, employment type, teaching responsibilities, and weekly working hours. Categorical variables were converted into dummy variables by selecting a reference group for each variable and assigning binary codes (1 or 0) to other categories. The categorical variables and their reference groups were defined as follows: age (<30 years, 30–34 years, 35–39 years, 40–44 years, 45–49 years, ≥50 years; reference group: <30 years); education (bachelor’s degree, postgraduate degree, other; reference group: bachelor’s degree); marital status (unmarried, married; reference group: unmarried); hospital tier (Tertiary Grade A, Tertiary Grade B, secondary hospitals; reference group: Tertiary Grade A); professional qualifications (physician, attending physician, associate chief physician, chief physician; reference group: physician); administrative role (holding administrative duties, not holding administrative duties; reference group: holding administrative duties); employment type (authorized personnel, contract personnel; reference group: authorized personnel); teaching responsibilities (undertaking teaching responsibilities, not undertaking teaching responsibilities; reference group: undertaking teaching responsibilities); weekly working hours (<40 h, 40–60 h, >60 h; reference group: <40 h). A two-tailed *p* < 0.05 was considered statistically significant.

## 3 Results

### 3.1 Demographic characteristic

The demographic characteristics of the respondents are detailed in Table 1. The study sample was evenly distributed between males and females, with the majority of participants aged under 35 years. The educational background of the respondents was predominantly at the bachelor’s and master’s levels, and their positions were mainly junior physicians and attending physicians, with a significant proportion holding senior titles such as deputy chief physician and chief physician. Marital status and administrative distribution were consistent with the age and professional title demographics, aligning with the situation. Most of the surveyed individuals were anesthesiologists from tertiary hospitals, with 92% working more than 40 h per week. Additionally, 75.2% of the physicians undertook teaching responsibilities alongside their clinical duties.

**Table 1:** Description of participant characteristics.

Variable		Analysis sample (n)	Proportion (%)
Gender	Male	155	47.5
	Female	171	52.5
Age	<30	179	54.9
	30 ~ 34	86	26.4
	35 ~ 39	19	5.8
	40 ~ 44	14	4.3
	45 ~ 49	12	3.7
	≥50	16	4.9
Education	Undergraduate	97	29.8
	Postgraduate	220	67.5
	Other	9	2.8
Marital status·	Single	208	63.8
	Married	118	36.2
Professional qualifications	Resident physicians	245	75.2
	Attending doctor	53	16.3
	Deputy chief physician	22	6.7
	Chief physician	6	1.8
Working hours per week	<40 h	26	8.0
	40–60 h	213	65.3
	>60 h	87	26.7
Hospital level	Tertiary hospital (Grade a)	258	79.1
	Tertiary hospital (Grade b)	56	17.2
	Secondary hospital	12	3.7
Employment form	Authorized personnel	108	33.1
	Contract personnel	218	66.9
Administrative function	True	86	26.4
	False	240	73.6
Teaching task	True	245	75.2
	False	81	24.8

Data are expressed as the number of samples in different categories (*n*) and the percentage of the total sample size (%) to show the basic information of the 326 subjects who participated in this survey. The data represent the number of samples (*n*) in different categories and the percentage (%) of the total sample size, illustrating the basic information of the 326 participants in this survey.

### 3.2 Current status analysis of job burnout

**Figure 1:**
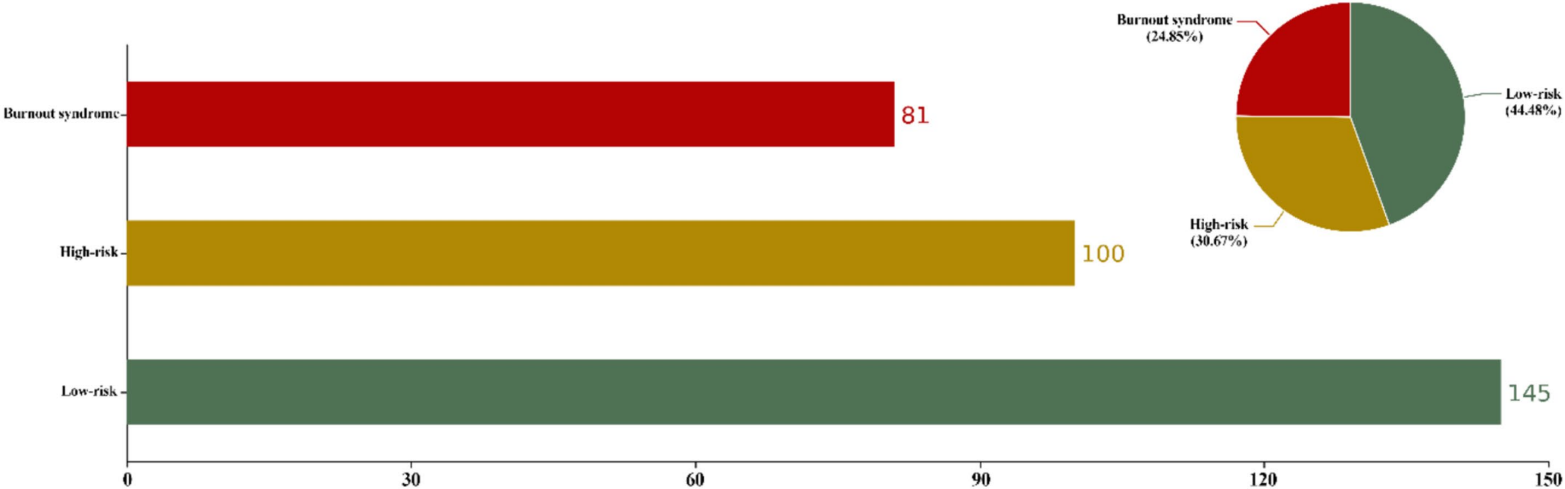
Degree of job burnout. Based on the 326 responses, the rate of the degree of burnout among anesthesiologists is shown. EE (≥27) and/or DP (≥10) were considered to be at high risk of burnout. If there is PA (≤33), it is considered job burnout syndrome. Those who do not meet the above criteria are classified as low risk of job burnout. Color-coded legend: Red: High-risk burnout (EE ≥ 27 or DP ≥ 10). Claybank: Burnout syndrome PA ≤ 33 based on EE ≥ 27 or DP ≥ 10. Green: Low-risk (no criteria met).

Within individual dimensions, the prevalence rates were 27.91% for high emotional exhaustion (EE ≥ 27), 53.99% for depersonalization (DP ≥ 10), and 89.88% for a low sense of personal accomplishment (PA ≤ 33). Notably, over half of the anesthesiologists exhibited depersonalization and a low sense of accomplishment. The proportion of anesthesiologists at high risk of job burnout was 30.67%, with 24.85% meeting the criteria for burnout syndrome, culminating in a combined proportion of 55.52% (Table 2 and Figure 1).

**Table 2:** Job burnout dimension scores.

Dimension	Analysis sample (*n* = 326)	Proportion %
Emotional exhaustion	91	27.91
Depersonalization	176	53.99
Low sense of personal accomplishment	293	89.88

The data represent the proportion (%) of the 326 subjects participating in this survey in the three dimensions of emotional exhaustion, depersonalization, and low personal accomplishment, respectively, to show the overall job burnout of the research population. Emotional exhaustion: emotional exhaustion ≥27 indicated. Depersonalization: depersonalization ≥10 indicated. Low sense of accomplishment: personal sense of accomplishment ≤33.

### 3.3 Univariate analysis of high risk of burnout, psychological status, and job satisfaction

This study identified significant associations between educational level and EE (Eta-squared = 0.079, 95% CI [0.029 ~ 0.136], *p* < 0.001) and DP (Eta-squared = 0.106, 95% CI [0.048 ~ 0.169], *p* < 0.001). Anesthesiologists in tertiary hospitals reported higher EE (Eta-squared = 0.084, 95% CI [0.032 ~ 0.142], *p* < 0.001) and DP (Eta-squared = 0.098, 95% CI [0.042 ~ 0.159], *p* < 0.001), with significant differences observed compared to those in hospitals with lower accreditation levels. Contract staff had the EE and DP higher scores (EE: Cohen’s *d* = −0.581, 95% CI [−0.816 ~ −0.346], *p* < 0.001); (DP: Cohen’s *d* = −0.643, 95% CI [−0.878 ~ −0.407], *p* < 0.001). In terms of age, individuals over 35 years old scored lower on DP than those under 35 (Eta-squared = 0.109, 95% CI [0.043 ~ 0.164], *p* < 0.001) (Table 3).

**Table 3:** Univariate analysis of high risk of burnout.

Variable		MBI-HSS											
		EE				DP				PA			
		Mean ± SD	*P*-value	Effect size	95%CI	Mean ± SD	*P*-value	Effect size	95%CI	Mean ± SD	*P*-value	Effect size	95%CI
Gender	Male	2.18 ± 1.030	0.479	−0.079	(−0.296 ~ 0.139)	1.95 ± 1.07	0.313	−0.112	(−0.330 ~ 0.105)	2.79 ± 1.19	0.782	−0.031	(−0.248 ~ 0.187)
	Female	2.26 ± 0.97				2.06 ± 0.98				2.83 ± 1.07			
Age	<30	2.37 ± 1.02	0.014*	0.043	(0.002 ~ 0.08)	2.21 ± 0.96	<0.001**	0.109	(0.043 ~ 0.164)	2.72 ± 1.00	<0.001**	0.120	(0.051 ~ 0.177)
	30 ~ 34	2.15 ± 1.00				2.05 ± 1.07				2.46 ± 1.10			
	35 ~ 39	1.98 ± 0.95				1.64 ± 0.90				3.51 ± 1.21			
	40 ~ 44	2.19 ± 0.81				1.39 ± 0.85				3.43 ± 0.89			
	45 ~ 49	1.45 ± 0.60				1.08 ± 0.68				3.72 ± 1.35			
	≥50	1.9 ± 0.89				1.16 ± 0.98				3.72 ± 1.38			
Education	Undergraduate	1.80 ± 0.91	<0.001**	0.079	(0.029 ~ 0.136)	1.61 ± 0 0.98	<0.001**	0.106	(0.048 ~ 0.169)	2.94 ± 1.31	0.001*	0.041	(0.007 ~ 0.088)
	Postgraduate	2.42 ± 0.98				2.23 ± 0.97				2.71 ± 1.00			
	Other	1.98 ± 0.93				0.96 ± 0.94				4.01 ± 1.15			
Marital status	Single	2.32 ± 1.01	0.017*	0.276	(0.049 ~ 0.503)	2.20 ± 0.96	<0.001**	0.530	(0.300 ~ 0.759)	2.58 ± 1.02	<0.001**	−0.600	(−0.830 ~ −0.369)
	Married	2.05 ± 0.95				1.67 ± 1.06				3.23 ± 1.19			
Professional qualifications	Resident physicians	2.53 ± 1.01	<0.001**	0.055	(0.012 ~ 0.103)	2.21 ± 0.99	<0.001**	0.122	(0.058 ~ 0.185)	2.65 ± 1.00	<0.001**	0.065	(0.018 ~ 0.117)
	Attending doctor	1.76 ± 0.94				1.49 ± 0.93				3.23 ± 1.45			
	Deputy chief physician	2.08 ± 0.76				1.35 ± 0.85				3.31 ± 1.11			
	Chief physician	1.63 ± 0.59				0.80 ± 0.59				3.83 ± 1.13			
Working hours per week	<40 h	1.48 ± 0.64	<0.001**	0.087	(0.035 ~ 0.146)	1.42 ± 0.68	<0.001**	0.071	(0.024 ~ 0.126)	2.56 ± 1.40	0.292	0.008	(0.000 ~ 0.033)
	40–60 h	2.16 ± 0.95				1.92 ± 1.01				2.88 ± 1.16			
	≥60 h	2.61 ± 1.04				2.40 ± 1.02				2.73 ± 0.95			
Hospital level	Tertiary hospital (Grade a)	2.37 ± 0.99	<0.001**	0.084	(0.032 ~ 0.142)	2.16 ± 1.00	<0.001**	0.098	(0.042 ~ 0.159)	2.79 ± 1.03	0.006*	0.032	(0.003 ~ 0.074)
	Tertiary hospital (Grade b)	1.71 ± 0.83				1.51 ± 0.90				2.71 ± 1.30			
	Secondary hospital	1.46 ± 0.80				0.95 ± 0.77				3.82 ± 1.77			
Employment Form	Authorized personnel	1.85 ± 0.87	<0.001**	−0.581	(−0.816 ~ −0.346)	1.59 ± 0.93	<0.001**	−0.643	(−0.878 ~ −0.407)	2.87 ± 1.35	0.553	0.077	(−0.154 ~ 0.308)
	Contract personnel	2.41 ± 1 0.01				2.22 ± 1.01				2.78 ± 1.00			
Administrative function	True	1.83 ± 0.94	<0.001**	−0.552	(−0.801 ~ −0.302)	1.62 ± 0.99	<0.001**	−0.528	(−0.778 ~ −0.278)	2.43 ± 1.29	<0.001**	−0.469	(−0.718 ~ −0.220)
	False	2.36 ± 0.98				2.15 ± 1.00				2.95 ± 1.04			
Teaching task	True	2.29 ± 1.00	0.045*	0.258	(0.006 ~ 0.510)	2.09 ± 1.01	0.013*	0.320	(0.067 ~ 0.572)	2.74 ± 1.07	0.052	−0.249	(−0.501 ~ −0.003)
	False	2.03 ± 0.98				1.76 ± 1.03				3.02 ± 1.26			

Values are expressed as mean ± SD. An independent samples *t*-test was used for comparisons between two groups, with the effect size being Cohen’s *d* and its 95% confidence interval. An analysis of variance (ANOVA) test was employed for comparisons among multiple groups, with the effect size being Eta-squared and its 95% confidence interval. Cohen’s *d* and Eta-squared are uniformly referred to as effect size in this table. *p*-values are bolded and indicate statistical significance, with *P* < 0.05* and *P* < 0.001**. MBI-HSS, Maslach Burnout Inventory-Human Services Survey; EE, emotional exhaustion; DP, depersonalization; PA, personal accomplishment.

In this study, age was found to be a significant factor affecting depression, anxiety, and stress levels (Eta-squared = 0.084, 95% CI [0.025 ~ 0.133], *p* < 0.001; Eta-squared = 0.106, 95% CI [0.041 ~ 0.161], *p* < 0.001; Eta-squared = 0.073, 95% CI [0.018 ~ 0.120], *p* < 0.001), with the 30–34 age group exhibiting the highest scores for depression and anxiety. Regarding educational attainment, individuals with a master’s degree reported higher levels of depression and anxiety (Eta-squared = 0.075, 95% CI [0.027 ~ 0.132], *p* < 0.001; Eta-squared = 0.072, 95% CI [0.025 ~ 0.128], *p* < 0.001) compared to those with a bachelor’s degree. There were also differences in depression and anxiety levels among professionals with varying job titles, with junior doctors showing higher depression scores (Eta-squared = 0.053 95% CI [0.011 ~ 0.100], *p* < 0.001). Furthermore, longer weekly working hours were associated with higher stress scores (Cohen’s *d* = 0.057, 95% CI [0.016 ~ 0.109], *p* < 0.001) (Table 4).

**Table 4:** Univariate analysis of psychological status.

Variable		DASS-21											
		Depression				Anxiety				Stress			
		Mean ± SD	*P*-value	Effect size	95%CI	Mean ± SD	*P*-value	Effect size	95%CI	Mean ± SD	*P*-value	Effect size	95%CI
Gender	Male	9.08 ± 6.68	0.192	0.145	(−0.073 ~ 0.363)	9.07 ± 7.13	0.192	0.145	(−0.073 ~ 0.362)	10.3 ± 7.11	0.003*	0.303	(0.084 ~ 0.521)
	Female	8.13 ± 6.40				8.08 ± 6.53				8.29 ± 6.15			
Age	<30	7.73 ± 5.74	<0.001**	0.084	(0.025 ~ 0.133)	7.49 ± 5.97	<0.001**	0.106	(0.041 ~ 0.161)	7.91 ± 6.05	<0.001**	0.073	(0.018 ~ 0.120)
	30 ~ 34	11.55 ± 7.77				12.08 ± 7.88				11.65 ± 7.16			
	35 ~ 39	5. 11 ± 5.16				5.05 ± 5.59				7.47 ± 7.74			
	40 ~ 44	7.71 ± 4.14				8.71 ± 6.06				12.71 ± 6.54			
	45 ~ 49	6.83 ± 7.06				5.83 ± 5.36				9.50 ± 5.66			
	≥50	8.38 ± 5.90				7.5 ± 6.30				10.13 ± 6.39			
Education	Undergraduate	5.82 ± 5.47	<0.001**	0.075	(0.027 ~ 0.132)	5.75 ± 5.34	<0.001**	0.072	(0.025 ~ 0.128)	6.97 ± 6.40	<0.001**	0.052	(0.013 ~ 0.102)
	Postgraduate	9.75 ± 6.69				9.79 ± 7.10				10.13 ± 6.61			
	Other	9.56 ± 5.27				8.44 ± 5.98				12.22 ± 5.95			
Marital status	Single	9.09 ± 6.41	0.061	0.217	(−0.010 ~ 0.443)	9.01 ± 6.54	0.105	0.187	(−0.039 ~ 0.414)	9.35 ± 6.53	0.359	0.042	(−0.184 ~ 0.267)
	Married	7.68 ± 6.71				7.74 ± 7.27				9.07 ± 6.99			
Professional qualifications	Resident physicians	9.39 ± 6.75	<0.001**	0.053	(0.011 ~ 0.100)	9.32 ± 7.06	0.002*	0.046	(0.007 ~ 0.091)	9.48 ± 6.76	0.116	0.018	(0.000 ~ 0.0)9
	Attending doctor	5.45 ± 5.50				6.09 ± 5.75				7.58 ± 6.76			
	Deputy chief physician	7.91 ± 4. 30				7.55 ± 5.12				11.18 ± 5.61			
	Chief physician	5.67 ± 5.13				2.67 ± 2.42				7.33 ± 4.13			
Working hours per week	<40 h	5.65 ± 5.52	<0.001**	0.045	(0.009 ~ 0.093)	6.23 ± 5.72	0.012**	0.027	(0.001 ~ 0.067)	5.77 ± 5.26	<0.001**	0.057	(0.016 ~ 0.109)
	40–60 h	8.10 ± 6.29				8.16 ± 6.69				8.73 ± 6.3			
	≥60 h	10.62 ± 6.96				10.2 ± 7. 19				11.54 ± 7.32			
Hospital level	Tertiary hospital (Grade a)	9.29 ± 6.62	<0.001**	0.045	(0.009 ~ 0.092)	9.23 ± 7.01	0.002*	0.038	(0.006 ~ 0.083)	9.78 ± 6.74	0.012*	0.027	(0.001 ~ 0.067)
	Tertiary hospital (Grade b)	5.98 ± 5.84				5.93 ± 5.57				6.86 ± 6.17			
	Secondary hospital	5.50 ± 3.92				6.17 ± 4.93				8.83 ± 5.60			
Employment form	Authorized personnel	7.94 ± 6.84	0.211	−0.147	(−0.378 ~ 0.084)	8. 16 ± 6.72	0.464	−0.086	(−0.317 ~ 0.144)	8.74 ± 6.76	0.338	−0.113	(−0.344 ~ 0.118)
	Contract personnel	8.90 ± 6.39				8.75 ± 6.89				9.50 ± 6.65			
Administrative function	True	10.16 ± 7.07	0.004*	0.331	(0.083 ~ 0.579)	10.74 ± 7.2	<0.001**	0.443	(0.194 ~ 0.692)	11.05 ± 6.3	0.002*	0.370	(0.122 ~ 0.618)
	False	8.01 ± 6.27				7.77 ± 6.53				8.60 ± 6.72			
Teaching task	True	8.92 ± 6.43	0.100	0.211	(−0.041 ~ 0.463)	8.89 ± 6.85	0.121	0.199	(−0.052 ~ 0.451)	9.50 ± 6.62	0.229	0.155	(−0.097 ~ 0.406)
	False	7.54 ± 6.82				7.53 ± 6.71				8.47 ± 6.88			

Values are expressed as mean ± SD. An independent samples *t*-test was used for comparisons between two groups, with the effect size being Cohen’s *d* and its 95% confidence interval. An analysis of variance (ANOVA) test was employed for comparisons among multiple groups, with the effect size being Eta-squared and its 95% confidence interval. Cohen’s *d* and Eta-squared are uniformly referred to as effect size in this table. *P*-values are bolded and indicate statistical significance, with *P* < 0.05* and *P* < 0.001**. DASS-21, Depression Anxiety and Stress Scale – 21 Items.

The study findings indicate anesthesiologists employed at tertiary level A hospitals reported significantly lower general (Eta-squared = 0.032, 95% CI [0.003 ~ 0.074], *p* < 0.05) and internal satisfaction (Eta-squared = 0.042, 95% CI [0.007 ~ 0.088], *p* < 0.05) than those at tertiary level B hospitals. There was also a significant variation in general (Eta-squared = 0.047, 95% CI [(0.008 ~ 0.092)], *p* < 0.05) and internal satisfaction (Eta-squared = 0.087, 95% CI [0.032 ~ 0.144], *p* < 0.001) across different job titles, with junior doctors recording the lowest levels of job satisfaction. Job satisfaction was negative with longer weekly working hours (Eta-squared = 0.069, 95% CI [0.023 ~ 0.124], *p* < 0.001) (Table 5).

**Table 5:** Univariate analysis of job satisfaction.

Variable		MSQ-SF											
		General				Internal				External			
		Mean ± SD	*P*-value	Effect size	95%CI	Mean ± SD	*P*-value	Effect size	95%CI	Mean ± SD	*P*-value	Effect size	95%CI
Gender	Male	3.60 ± 0.51	0.235	0.132	(−0.086 ~ 0.349)	3.67 ± 0.52	0.118	0.174	(−0.044 ~ 0.391)	3.46 ± 0.66	0.928	0.010	(−0.207 ~ 0.227)
	Female	3.53 ± 0.50				3.58 ± 0.49				3.46 ± 0.60			
Age	<30	3.51 ± 0.49	0.168	0.024	(0.000 ~ 0.051)	3.54 ± 0.47	0.002*	0.056	(0.008 ~ 0.098)	3.43 ± 0.59	0.755	0.008	(0.000 ~ 0.02)
	30 ~ 34	3.59 ± 0.54				3.61 ± 0.55				3.55 ± 0.61			
	35 ~ 39	3.64 ± 0.61				3.79 ± 0.54				3.39 ± 0.80			
	40 ~ 44	3.76 ± 0.60				3.89 ± 0.51				3.51 ± 0.83			
	45 ~ 49	3.78 ± 0.52				3.96 ± 0.51				3.49 ± 0.73			
	≥50	3.68 ± 0.43				3.83 ± 0.34				3.40 ± 0.71			
Education	Undergraduate	3.67 ± 0.52	0.032*	0.021	(0.000 ~ 0.058)	3.76 ± 0.49	0.002*	0.038	(0.006 ~ 0.083)	3.51 ± 0.67	0.60	0.003	(0.000 ~ 0.021)
	Postgraduate	3.52 ± 0.51				3.55 ± 0.51				3.44 ± 0.60			
	Other	3.69 ± 0.46				3.81 ± 0.37				3.41 ± 0.77			
Marital status	Single	3.52 ± 0.49	0.031*	−0.250	(−0.477 ~ 0.023)	3.55 ± 0.48	<0.001**	−0.391	(−0.618 ~ −0.163)	3.46 ± 0.60	0.944	−0.001	(−0.2226 ~ 0.225)
	Married	3.65 ± 0.55				3.74 ± 0.53				3.46 ± 0.69			
Professional qualifications	Resident physicians	3.50 ± 0.49	0.002*	0.047	(0.008 ~ 0.092)	3.53 ± 0.49	<0.001**	0.087	(0.032 ~ 0.144)	3.43 ± 0.59	0.410	0.009	(0.000 ~ 0.031)
	Attending doctor	3.77 ± 0.55				3.88 ± 0.48				3.58 ± 0.74			
	Deputy chief physician	3.73 ± 0.46				3.85 ± 0.41				3.53 ± 0.61			
	Chief physician	3.73 ± 0.69				3.96 ± 0.57				3.31 ± 0.99			
Working hours per week	<40 h	3.86 ± 0.47	<0.001**	0.069	(0.023 ~ 0.124)	3.89 ± 0.50	<0.001**	0.063	(0.019 ~ 0.117)	3.83 ± 0.50	<0.001**	0.068	(0.022 ~ 0.123)
	40–60 h	3.61 ± 0.50				3.66 ± 0.48				3.51 ± 0.61			
	≥60 h	3.37 ± 0.51				3.43 ± 0.51				3.23 ± 0.64			
Hospital level	Tertiary hospital (Grade a)	3.52 ± 0.50	0.006*	0.032	(0.003 ~ 0.074)	3.57 ± 0.50	0.001*	0.042	(0.007 ~ 0.088)	3.43 ± 0.61	0.192	0.010	(0.000 ~ 0.038)
	Tertiary hospital (Grade b)	3.72 ± 0.52				3.77 ± 0.48				3.58 ± 0.69			
	Secondary hospital	3.83 ± 0.58				3.99 ± 0.53				3.58 ± 0.78			
Employment Form	Authorized personnel	3.79 ± 0.52	<0.001**	0.684	(0.447 ~ 0.920)	3.87 ± 0.51	<0.001**	0.777	(0.538 ~ 1.014)	3.64 ± 0.67	<0.001**	0.441	(0.208 ~ 0.674)
	Contract personnel	3.46 ± 0.47				3.50 ± 0.46				3.37 ± 0.59			
Administrative function	True	3.77 ± 0.54	<0.001**	0.556	(0.306 ~ 0.806)	3.81 ± 0.54	<0.001**	0.517	(0.267 ~ 0.766)	3.72 ± 0.64	<0.001**	0.572	(0.321 ~ 0.822)
	False	3.49 ± 0.49				3.55 ± 0.48				3.37 ± 0.60			
Teaching task	True	3.56 ± 0.52	0.747	−0.041	(−0.293 ~ 0.21)	3.61 ± 0.51	0.524	−0.082	(−0.333 ~ 0.17)	3.47 ± 0.62	0.744	0.042	(−0.209 ~ 0.293)
	False	3.58 ± 0.51				3.65 ± 0.48				3.44 ± 0.66			

Values are expressed as mean ± SD. An independent samples *t*-test was used for comparisons between two groups, with the effect size being Cohen’s *d* and its 95% confidence interval. An analysis of variance (ANOVA) test was employed for comparisons among multiple groups, with the effect size being Eta-squared and its 95% confidence interval. Cohen’s *d* and Eta-squared are uniformly referred to as effect size in this table. *P*-values are bolded and indicate statistical significance, with *P* < 0.05* and *P* < 0.001**. MSQ-SF, Minnesota Satisfaction Questionnaire-Short Form; General, general satisfaction; Internal, internal satisfaction; External, external satisfaction.

### 3.4 Correlation analysis of job burnout, psychological status and satisfaction

**Figure 2:**
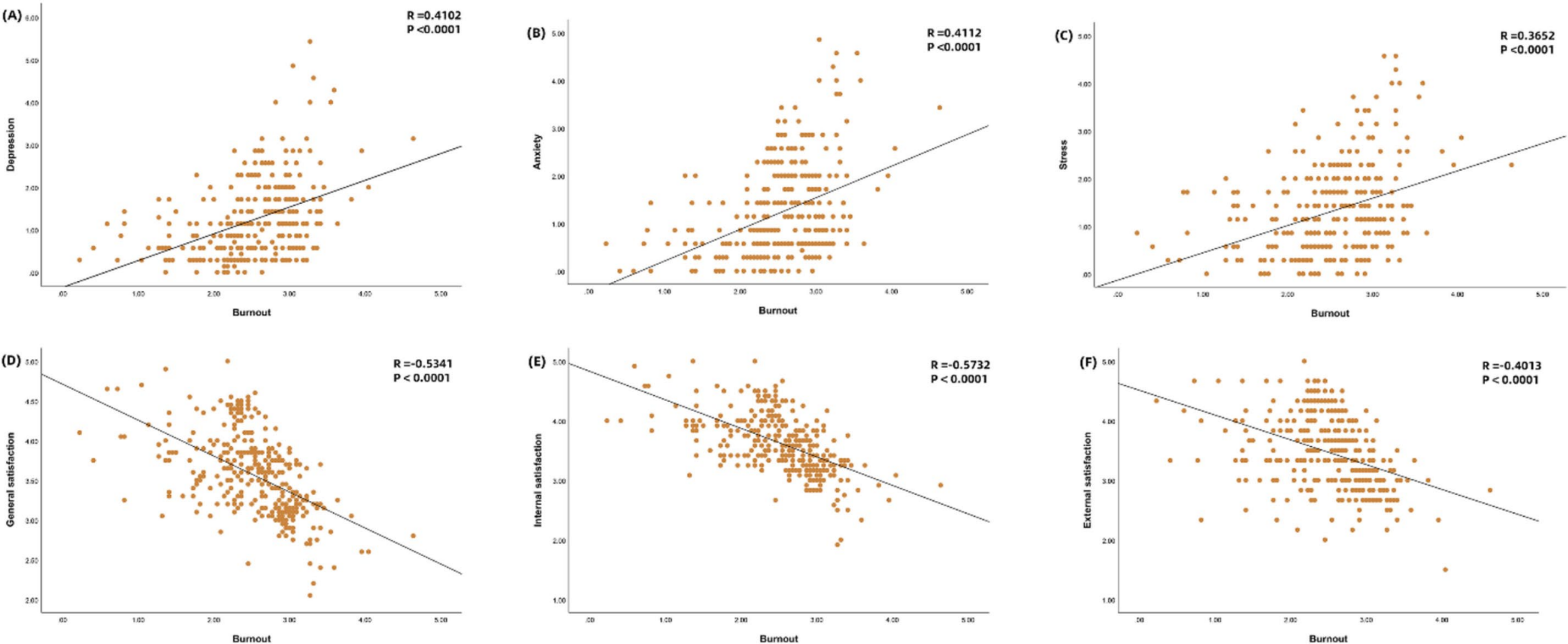
Correlation of job burnout with psychological status and job satisfaction. Person correlation analysis was used. Burnout and depression **(A)**, burnout and anxiety **(B)**, burnout and stress **(C)**, burnout and general satisfaction **(D)**, burnout and intrinsic satisfaction **(E)**, and burnout and extrinsic satisfaction **(F)**.

The study demonstrated a significant positive correlation between the three dimensions of psychological status and burnout (*p* < 0.001), with poorer psychological states associated with higher levels of burnout. Conversely, a significant negative correlation was observed between the three dimensions of job satisfaction and burnout (*p* < 0.001), indicating that increased job satisfaction is linked to reduced burnout. These relationships are visualized in Figure 2.

After adjusting for demographic variables, a multiple linear regression analysis was performed. The VIF values of the five independent variables involved in this study are all less than 5, indicating no multicollinearity among the independent variables (Table 6). The anxiety and stress components of the psychological assessment significantly and positively predicted burnout among anesthesiologists, with beta coefficients of 0.313 (95% CI = 0.064–0.562, *p* = 0.014) and 0.253 (95% CI = 0.007–0.499), respectively. It suggests that for each one-unit increase in anxiety or stress, burnout increases by 0.313 or 0.253 units. Internal and extrinsic satisfaction significantly and negatively predicted burnout, with beta coefficients of −0.566 (95% CI = −0.870–−0.262, *p* < 0.001) and-0.475 (95% CI = −0.938–−0.011, *p* = 0.045), respectively. It implies that burnout decreases by 0.566 or 0.475 units for each one-unit increase in satisfaction. Collectively, these predictors accounted for 55.00% of the variance in burnout among anesthesiologists.

**Table 6:** Correlation analysis of job burnout, psychological status and satisfaction.

Variable	*B*	Standard error	Beta	*B* of 95%Cl		*t*	*P*	VIF
				Lower bound	Upper bound			
(Constant)	88.786	5.402	–	76.156	97.416	16.066	<0.001**	–
Depression	0.035	0.138	0.017	−0.237	0.306	0.253	0.801	3.316
Anxiety	0.313	0.126	0.160	0.064	0.562	2.447	0.014*	3.029
Stress	0.253	0.125	0.127	0.007	0.499	2.024	0.044*	2.840
Internal satisfaction	−0.566	0.154	−0.258	−0.870	−0.262	−3.666	<0.001**	3.575
External satisfaction	−0.475	0.236	−0.134	−0.938	−0.011	−2.014	0.045*	3.208
	Adjusted R Square	0.550						
	F	18.290						
	P	<0.001**						
Dependent variable: Burnout								

The data presents the results of a multiple linear regression analysis of job burnout with psychological state and satisfaction. Job burnout is the dependent variable, while the various dimensions of DASS-21 and job satisfaction are the independent variables. Age, education, marital status, hospital level, professional qualifications, administrative function, employment form, teaching task, and working hours per week are covariates. B represents the unstandardized regression coefficient, and Beta represents the standardized regression coefficient. VIF can be used to determine multicollinearity; when VIF < 5, there is no multicollinearity among the independent variables. Adjusted *R* Square indicates that the model can explain 55% of the variance. *P*-values are bolded and are statistically significant, with *P* < 0.05* and *P* < 0.001**.

## 4 Discussion

This study reveals a high prevalence of occupational burnout among Chinese anesthesiologists in the post-COVID-19 era (55.52%), with 30.67% meeting burnout criteria and 24.85% diagnosed with burnout syndrome. Compared to pre-COVID-19 era studies in China, occupational burnout among anesthesiologists in this study has significantly increased (2). Meanwhile, Afonso et al. (7) reported that 18.9% of U. S. anesthesiologists exhibited burnout syndrome in the post-COVID-19 era, slightly lower than the findings here. This discrepancy may be linked to the high-stress environment Chinese anesthesiologists face the post-COVID-19 era. The healthcare industry is characterized by high demands, low control, and elevated burnout (16). In the post-COVID-19 era, increased societal employment pressure intensified industry competition, and surging surgical volumes have frequently forced anesthesiologists to confront higher-intensity, high-risk workloads. Physical and mental health deteriorate when working hours and demands exceed human limits (6, 17). In this study, 92% of participants worked over 40 h weekly, exhibiting higher burnout levels. International studies corroborate that prolonged working hours correlate with increased burnout, indicating a threshold beyond which human capacity declines (17, 18). Notably, anesthesiologists in tertiary hospitals, particularly those with postgraduate degrees and contract-based employment, faced higher burnout. Li et al. (3) argue that China’s uneven healthcare system distribution, age, hospital tier, and working hours are critical burnout determinants. Western studies emphasize this trend less, likely reflecting China’s unique medical system. Additionally, our study revealed no significant correlation between gender and burnout among anesthesiologists, a finding that diverges from previous research. Prior studies have indicated that male surgeons are less likely to experience burnout than female surgeons (19). However, some studies have identified a relationship between pathological personality traits and burnout in male physicians, suggesting that specific personality characteristics may predispose male doctors to higher levels of professional burnout (20). We suggest that this difference may stem from changing work patterns in the post-COVID-19 era, in contrast to the context of previous studies, suggesting that gender is not the only independent risk factor for developing burnout. Furthermore, there may be complex interactions at play that could influence the development of burnout. In light of this, our study refrains from drawing overly broad conclusions regarding the relationship between gender and burnout, pending further research that validates and elucidates these interactions.

Anesthesiologists often face physical or mental health problems due to excessive workload, interpersonal management, fear of medical litigation, and sleep deprivation (11, 21, 22). In the post-COVID-19 era, anesthesiologists in high-stress environments will likely experience more negative emotions in their work. Our study confirmed that the emotional exhaustion and depersonalization scores of anesthesiologists in tertiary hospitals were significantly higher than those in secondary hospitals, reflecting the impact of occupational stress on mental health after the post-COVID-19 era. Notably, longer working hours were positively associated with higher emotional exhaustion and depersonalization scores. However, there were no significant differences in personal achievement. The study indicated that the sense of achievement of anesthesiologists is not solely affected by working hours, which warrants further investigation. In addition, this study found that anesthesiologists aged 30–34 years exhibited higher levels of anxiety and depression than other age groups. This group is the department’s backbone and is under more significant work stress and clinical responsibility. At the same time, the transformation of the medical system after the post-COVID-19 era amplifies the traditional pressure sources and gives rise to new challenges. In order to promote the development of the scientific medical system, the rapid popularization of digital medical treatment intensifies the pressure of technology transformation of this group. However, these psychological symptoms tend to improve in the older age group, which is consistent with the research results at home and abroad (23, 24). This phenomenon can be attributed to doctors in the elder group no longer facing the dual pressure of career rise and technical adaptation, and their work and life are less affected by the post-COVID-19 era. This study identifies negative correlations between burnout, depressive anxiety symptoms, and job satisfaction, consistent with Shanafelt et al.’s (25) findings in U. S. anesthesiologists. However, in China, anesthesiologists in tertiary hospitals, junior positions, and those working longer hours reported lower satisfaction. In the post-COVID-19 era, these groups faced precarious job security alongside unrelenting workloads, intensifying burnout and negative emotions, thereby reducing satisfaction.

The above analysis found significant differences between different education levels, hospital grades, administrative roles, and working hours in the dimensions of burnout, psychological status, and job satisfaction. Therefore, we sought to explore the interrelationship between these factors in the post-COVID-19 era and found that anesthesiologists with poorer psychological status mostly showed more severe burnout. The research of Zisook et al. (26) and Menon et al. (27) also established correlations among burnout, depression, and suicidal ideation. Although our data suggest a co-occurrence of burnout, physical health issues, and mental health problems that may collectively impact health-related quality of life, they do not clarify the causal relationship between burnout and depression. The overlap between certain aspects of burnout, particularly those concerning emotional expression, and the DASS-21 scores is expected. However, DASS-21 outcomes can only suggest the presence of negative emotions and are not diagnostic of depressive or anxiety disorders, necessitating further research to elucidate the relationship. It is crucial to differentiate between ‘burnout’ and ‘depression’; the former characterizes a crisis in one’s work-related identity, while the latter is a clinical syndrome (28–30). This study also confirmed (Figure 2) that there were instances of low mental status scores but relatively high burnout scores. This further suggests that mental health and burnout positively correlate but do not imply a causal relationship. Our study’s variability in individual responses highlights these associations’ complexity. More in-depth research is needed to explore the dynamic relationship between burnout and depressive anxiety emotions. In addition, our study revealed an inverse relationship between job satisfaction and burnout, a finding consistent with previous studies (28, 31). Specifically, as job satisfaction increases, burnout is relatively lower. This correlation suggests that a good work environment, support systems, and a sense of control and autonomy in professional roles contribute to an individual’s overall job satisfaction and can serve as protective factors against burnout, even in the high-stress environment of the post-COVID-19 era. Improving job satisfaction among anesthesiologists is key to preventing and managing burnout in the post-COVID-19 era.

The study comprehensively analyzed the complex relationship between job satisfaction, job burnout, and mental health of anesthesiologists in the post-COVID-19 era. The group with long working hours, poor mental status, and low job satisfaction had relatively higher job burnout. Based on the findings, we proposed a prospective intervention strategy. Young doctors should strengthen orientation training to cultivate inner satisfaction through mentorship programs and career growth opportunities, implement policies that limit the number of hours worked per week, establish accessible counseling and peer support networks, and advocate fair pay and career stability for informal staff. The study also has certain limitations. First, it proves a correlation, rather than a causal relationship, between job burnout and psychological status, and subsequent research should further investigate the complex interaction between the two. Second, the survey group is mainly concentrated in the southwest region, and the follow-up survey scope needs to be expanded to understand the job burnout of different regions. In summary, this study highlights the correlation among job burnout, mental health, and job satisfaction in anesthesiologists, which provides scientific theoretical support for improving the job well-being of anesthesiologists, a high-stress occupation.

## Data Availability

The raw data supporting the conclusions of this article will be made available by the authors, without undue reservation.

## Footnotes

^1^ https://www.wjx.cn
